# Frequent *SLC35A2* brain mosaicism in mild malformation of cortical development with oligodendroglial hyperplasia in epilepsy (MOGHE)

**DOI:** 10.1186/s40478-020-01085-3

**Published:** 2021-01-06

**Authors:** Thomas Bonduelle, Till Hartlieb, Sara Baldassari, Nam Suk Sim, Se Hoon Kim, Hoon-Chul Kang, Katja Kobow, Roland Coras, Mathilde Chipaux, Georg Dorfmüller, Homa Adle-Biassette, Eleonora Aronica, Jeong Ho Lee, Ingmar Blumcke, Stéphanie Baulac

**Affiliations:** 1 Institut du Cerveau - Paris Brain Institute - ICM, Inserm, CNRS, Sorbonne Université, Hôpital Pitié-Salpêtrière - 47, bd de l’hôpital, 75013 Paris, France; 2 Center for Pediatric Neurology, Neurorehabilitation and Epileptology, Schoen Klinik, Vogtareuth, Germany; 3 Paracelsus Medical University, Salzburg, Austria; 4 Graduate School of Medical Science and Engineering, KAIST, Daejeon, Korea; 5 Department of Pathology, Yonsei University College of Medicine, Seoul, Korea; 6 Division of Pediatric Neurology, Department of Pediatrics, Pediatric Epilepsy Clinics, Severance Children’s Hospital, Yonsei University College of Medicine, Seoul, Korea; 7 Department of Neuropathology, Universitätsklinikum Erlangen, Friedrich-Alexander University (FAU) Erlangen-Nürnberg, Erlangen, Germany; 8 Department of Pediatric Neurosurgery, Rothschild Foundation Hospital, 75019 Paris, France; 9 Université de Paris, service d’Anatomie Pathologique, AP-HP, Hôpital Lariboisière, DMU DREAM, UMR 1141, INSERM, Paris, France; 10 Department of (Neuro)Pathology, Amsterdam Neuroscience, Amsterdam UMC, University of Amsterdam, Amsterdam, The Netherlands; 11 Stichting Epilepsie Instellingen Nederland (SEIN), Heemstede, The Netherlands; 12 SoVarGen, Inc., Daejeon, 34051 Republic of Korea

**Keywords:** Malformations of cortical development, Epilepsy, Focal cortical dysplasia, *SLC35A2* gene, Glycosylation, Brain mosaicism

## Abstract

Focal malformations of cortical development (MCD) are linked to somatic brain mutations occurring during neurodevelopment. Mild malformation of cortical development with oligodendroglial hyperplasia in epilepsy (MOGHE) is a newly recognized clinico-pathological entity associated with pediatric drug-resistant focal epilepsy, and amenable to neurosurgical treatment. MOGHE is histopathologically characterized by clusters of increased oligodendroglial cell densities, patchy zones of hypomyelination, and heterotopic neurons in the white matter. The molecular etiology of MOGHE remained unknown so far. We hypothesized a contribution of mosaic brain variants and performed deep targeted gene sequencing on 20 surgical MOGHE brain samples from a single-center cohort of pediatric patients. We identified somatic pathogenic *SLC35A2* variants in 9/20 (45%) patients with mosaic rates ranging from 7 to 52%. *SLC35A2* encodes a UDP-galactose transporter, previously implicated in other malformations of cortical development (MCD) and a rare type of congenital disorder of glycosylation. To further clarify the histological features of *SLC35A2*-brain tissues, we then collected 17 samples with pathogenic *SLC35A2* variants from a multicenter cohort of MCD cases. Histopathological reassessment including anti-Olig2 staining confirmed a MOGHE diagnosis in all cases. Analysis by droplet digital PCR of pools of microdissected cells from one MOGHE tissue revealed a variant enrichment in clustered oligodendroglial cells and heterotopic neurons. Through an international consortium, we assembled an unprecedented series of 26 *SLC35A2*-MOGHE cases providing evidence that mosaic *SLC35A2* variants, likely occurred in a neuroglial progenitor cell during brain development, are a genetic marker for MOGHE.

**Electronic supplementary material:**

The online version of this article (10.1186/s40478-020-01085-3) contains supplementary material, which is available to authorized users.

## Introduction

Malformations of cortical development (MCD) represent an important cause of pediatric epilepsy resistant to anti-seizure medication [13, 37]. When focal, surgical resection of the malformed brain area currently represents the main treatment option for seizure control [3, 19]. Focal MCD comprise distinct histopathological entities, including Focal Cortical Dysplasia (FCD) type 1, 2 and 3 [4], mild MCD (mMCD), and a newly recognized clinico-pathological entity termed mild malformation of cortical development with oligodendroglial hyperplasia in epilepsy (MOGHE). MOGHE is histopathologically characterized by clusters of increased oligodendroglial cell density in the white matter and deep cortical layers, an augmented oligodendroglial proliferation activity, patchy areas with hypomyelination and an excess of heterotopic neurons in the white matter, which occurs most often in the frontal lobe of children with early seizure onset [35]. Magnetic resonance imaging (MRI) features of MOGHE are age-related and comprise increased laminar T2 and fluid attenuated inversion recovery (FLAIR) signal at the corticomedullary junction in young children (subtype I) and change to reduced corticomedullary differentiation due to increased signal of the adjacent white matter in older patients (subtype II) [16].

In recent years, somatic mosaicism has been shown to be a significant cause of MCD [23]. Brain mosaic variants in genes belonging to the mechanistic Target of Rapamycin (mTOR) pathway have been discovered as underlying cause of FCD type 2 [2, 8, 21, 26, 39]. Recently, mosaic variants in the *SLC35A2* gene, encoding the major Golgi‐localized UDP‐galactose transporter required for protein and sphingolipid glycosylation, have been identified in various forms of non-lesional focal epilepsies, as well as FCD type 1 and mMCD [2, 25, 38, 39, 41]. Moreover, germline de novo variants of *SLC35A2* cause a rare X-linked dominant form of congenital disorder of glycosylation (CDG) type IIm (MIM #300896), primarily presenting with epileptic encephalopathy, seizures, severe psychomotor developmental delay and delayed myelination [30, 40]. Loss of SLC35A2 protein function abolishes transport of UDP-galactose from the cytosol into the Golgi apparatus, resulting in the synthesis of truncated glycans lacking galactose residues [30, 38]; yet, how this relates to the clinical phenotype is still unknown.

In this study, we hypothesized that somatic brain variants in *SLC35A2* may contribute to the pathogenesis of MOGHE and used surgically resected brain tissues obtained from the epileptogenic zone to test this hypothesis. We found that mosaic *SLC35A2* variants are important factors in the etiology of MOGHE, and likely occurred in neuroglial progenitors during brain development.

## Materials and methods

### Cohort recruitment

We enrolled a cohort of 20 pediatric patients with refractory focal epilepsy subjected to surgery between 2012 and 2019 in the center for pediatric neurology, neurorehabilitation and epileptology, Schoen Klinik, Vogtareuth, Germany, and with a histopathological diagnosis of MOGHE. Eight out of twenty cases were previously reported [16]. All patients underwent detailed clinical examination and review of the medical files, including high-resolution magnetic resonance imaging (MRI), long-term video-EEG-monitoring (EEG) and fluoro-deoxy-glucose (FDG) positron emission tomography (PET) investigations when performed. Written informed consent was obtained from all participants or their parents on their behalf. The study was approved by the University of Erlangen ethical review board (EEBB 160_12B).

We next assembled an international cohort of 43 additional mMCD, FCD, unclassified FCD or inconclusive cases operated between 2013 and 2019 from three epilepsy surgery centers: the neurosurgery department from the Rothschild Foundation Hospital (n = 20, Paris, France), Severance Children’s Hospital (n = 17, Seoul, Republic of Korea) and Amsterdam UMC (n = 6, Amsterdam, the Netherlands). These studies received approval by the ethical committee CPP Ile de France II (No. ID-RCB/EUDRACT-2015-A00671-48), the Severance Hospital and KAIST Institutional Review Board and Committee on Human Research, and Amsterdam UMC (W13_85;W15_262).

### Tissues preparation and immunostainings in the 20 MOGHE cases

Resected brain specimens were formalin-fixed overnight in 4% formalin and processed into liquid paraffin according to standardized histopathology protocols [5]. All sections were cut at 4 μm, mounted on positively charged glass slides (Superfrost, Germany) and stained with hematoxylin and eosin (H&E) or Nissl-Luxol-Fast-Blue (Nissl-LFB). Immunohistochemical stainings were performed on selected slides followed by hematoxylin counterstaining. The following antibodies were used according to manufacturer’s protocols: NeuN (Clone A60; Millipore, Temecula, USA), MAP2 (clone HM-2, Sigma, St. Louis, USA), Mib1 (clone Ki67, cell marque, Rocklin, USA), Olig2 (clone JP18953, IBL International, Hamburg, Germany), and CNPase (clone 11-5B, Millipore).

### Neuropathological evaluation

The histopathology diagnosis of MOGHE was given according to the previously published catalogue of microscopic measures [35], i.e. clusters of oligodendroglial cells with high density at the grey-white matter junction using H&E staining and confirmed by additional immunohistochemistry for Olig2 and Mib1. Reassessment of the 43 multicentric mMCD cases was performed by an expert neuropathologist (IB) blind to the genetics results and the initial neuropathological diagnosis. Digitally scanned slides of H&E staining, NeuN and Olig2 immunostainings in all selected cases were made accessible for online microscopic evaluation (Leica Aperio, Germany).

### Gene panel sequencing

Genomic DNA from formalin-fixed paraffin-embedded (FFPE) brain tissues was extracted with QIAamp DNA Micro Kit (Qiagen) or Maxwell RSC DNA FFPE kit (Promega), following standard protocols. We designed a hybrid capture sequencing (Twist Bioscience), targeting coding exons and exon-flanking junctions (10 bp) of 59 genes including genes already involved in focal MCD, mTOR pathway-related genes and *SLC35A2* (the full list of genes included in the panel is reported in Supplemental data). Libraries were prepared according to a standard protocol for FFPE DNA samples and sequenced on an Illumina NextSeq 500 sequencer (2 × 75 bp) at the iGenSeq sequencing facility of ICM (Paris, France). Bioinformatic analysis including quality control, processing and calling of the variants was conducted by GenoSplice as previously described [2, 27]. FASTQ sequence files were mapped using hg19 reference with bwa-0.7.12 (mem) and converted to bam with samtools-1-1 (mpileup) to detect low frequency variants (i.e. < 10%). The pileup was parsed with pileup2base which provides the count of reads for each strand and nucleotides by coordinates. A home-made Perl script was developed to filter variants according to following criteria: (1) variants not retrieved by GATK; (2) ≥ 10 reads with the alternate allele; (3) ≥ 1% of allelic frequency; and (4) maximum strand bias value of 0.5. Candidate variants were validated by standard Sanger sequencing or deep site-specific amplicon sequencing. Variant pathogenicity for missense single nucleotide variant (SNV) was assessed using Mendelian Clinically Applicable Pathogenicity (M-CAP v1.4) [18] and Combined Annotation-Dependent Depletion (CADD v1.6) pathogenicity classifier website [7].

### Laser capture microdissection and droplet digital PCR

Laser capture microdissection (LCM) was performed on the frozen resected tissue of one MOGHE case (FR-2) subjected to surgery at the Rothschild Foundation Hospital (Paris, France). LCM was performed using a Leica LMD7000 system on 20 μm frozen brain sections mounted on PEN-membrane slides after rapid (30 s) cresyl-violet staining. Cell populations were selected as follows: (1) neurons: morphologically normal appearing neurons, oval shape, without Nissl substance cytoplasmic aggregates and longest cell body diameter *d* = 10–25 μm; (2) glial cells: small round-shaped cells, and *d* = 5–7 μm. We collected pools of 4 × 250 cortical normal neurons, 3 × 250 glial cells, and 3 × 70 heterotopic neurons from the subcortical white matter. To assess variant enrichment specifically in the oligodendroglial cell lineage, we performed a rapid immunostaining on a 20 μm frozen brain section mounted on a PEN-membrane slide using an anti-Olig2 primary antibody (clone EP112, Epitomics, 1:25) adapted from a previously published protocol [31]. We collected one pool of 250 and one pool of 500 Olig2-positive cells located in the regions of higher density within the white matter. Microdissected cells were collected in AdhesiveCap 500 Opaque tubes (Zeiss) for DNA extraction, as previously described [9]. We then performed droplet digital PCR (ddPCR) (QX200 system, Bio-Rad Laboratories), a highly sensitive technique based on sample partitioning following a Poisson distribution and allowing DNA absolute quantification, to detect specifically the variant in the different pools of cells. All reactions were prepared using the ddPCR Supermix for probes (no dUTPs) according the manufacturer’s protocol. Specific ddPCR Mutation Detection Assay (FAM + HEX) for the detection of *SLC35A2*:p.Ser212Leufs*9 variant was purchased from Bio-Rad. Data were analyzed using the QX200 droplet reader and Quantasoft Analysis Pro software version 1.0.596. The entire DNA extraction volume per pool of cells was used in each reaction.

### Statistical analysis

Genotype–phenotype correlations were conducted using the computing environment R provided by R Core Team (2019). Statistical analyses were performed using Chi-2 test to compare percentages from categorical variable and Wilcoxon-Mann–Whitney test to compare quantitative variable. Values of *p* < 0.05 were considered statistically significant.

## Results

### Neuropathological description of the MOGHE cohort

**Fig. 1:**
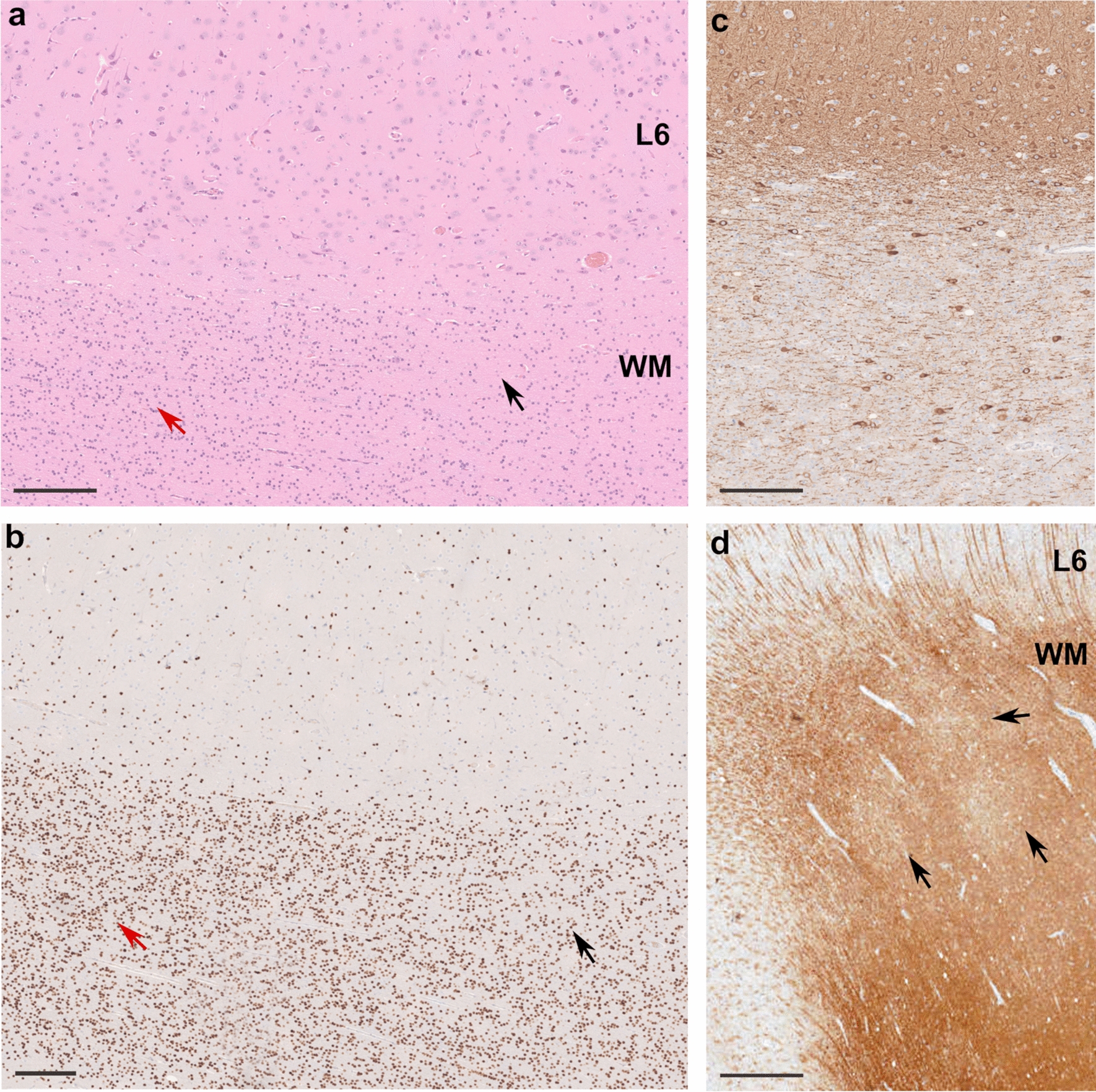
Representative histopathology findings in MOGHE. **a** H&E staining of the surgical tissue specimen of a 4-year old boy with frontal lobe epilepsy since age 3.3 years (patient DE-3 in Table 1) revealed clusters of increased oligodendroglial cell density (red arrow on left side). Normal oligodendroglial cell density is visible on the right (black arrow). L6: neocortical layer 6; WM: white matter (applies also to **b**). **b** Adjacent section stained with antibody against Olig2. Increased oligodendroglial cell density in WM (red arrow on the left) compared to normal density shown on right (black arrow) is visible. **c** Section stained with antibody against MAP2 indicating excessive heterotopic neurons in deep WM and grey-white matter junction. **d** Section stained with antibody directed against CNPase. Arrows indicate patchy areas with hypomyelination. Scale bar in a, b and c = 200 µm, in d = 1 mm

**Fig. 2:**
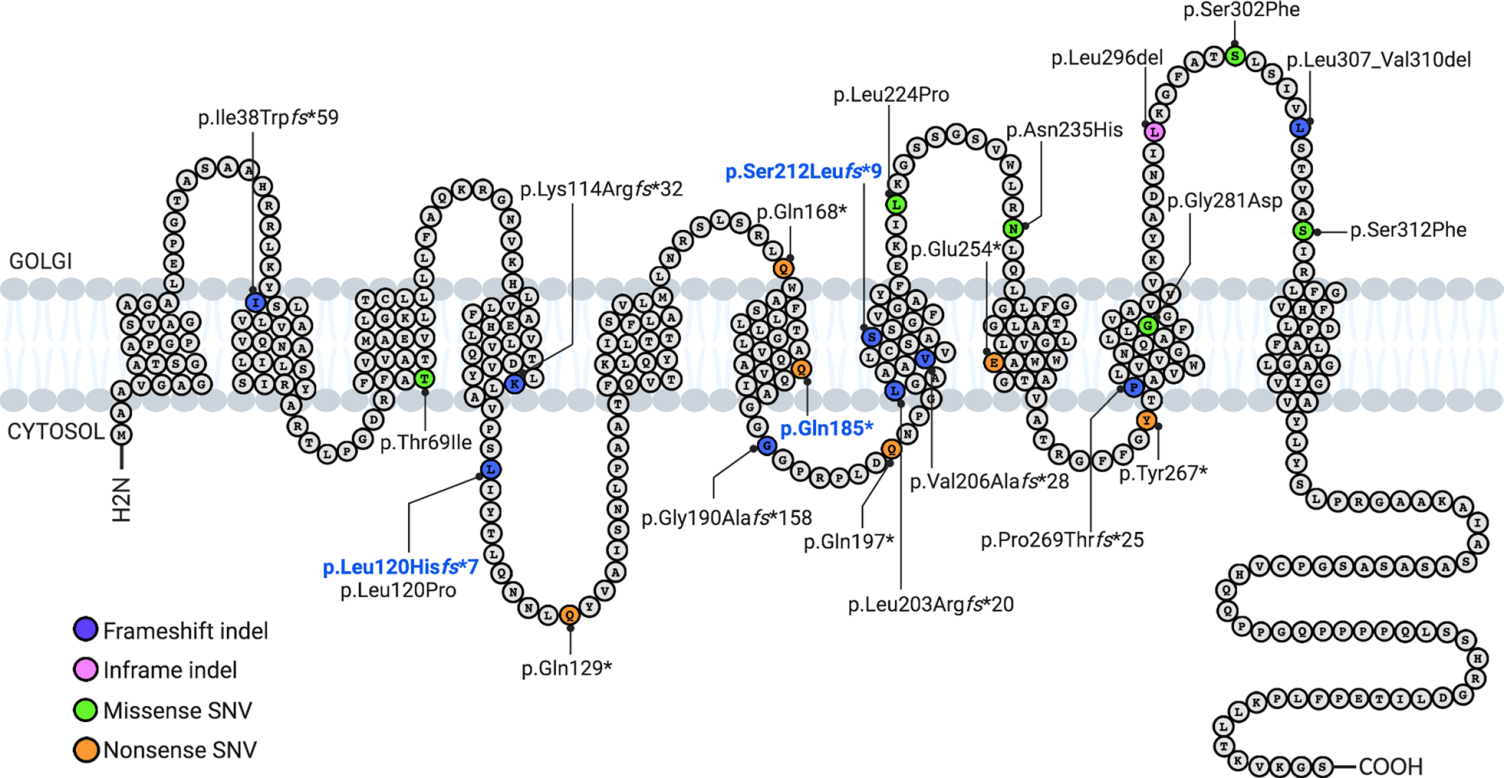
Position of MOGHE-related *SLC35A2* variants along the protein. SLC35A2 protein schematic showing even distribution of somatic variants identified in MOGHE patients. Somatic recurrent variants are highlighted in bold blue. *indel* insertion and/or deletion, *SNV* single nucleotide variant

**Fig. 3:**
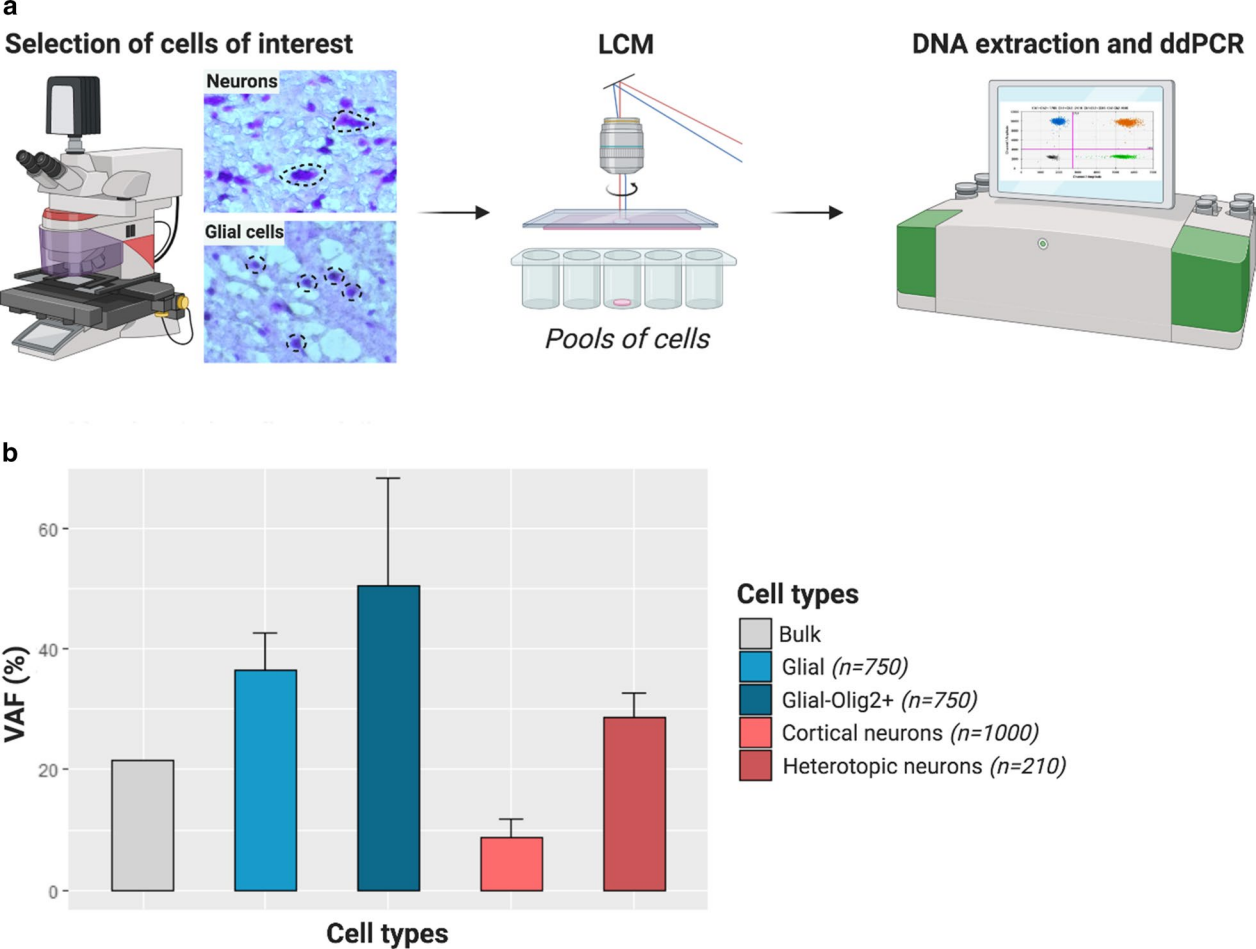
Olig2-positive cells and heterotopic neurons carry the pathogenic variants. **a** Cell populations were isolated by laser capture microdissection (LCM) based on their morphology. Droplet digital PCR (ddPCR) was used to detect the *SLC35A2* variant in pools of cells. **b** Barplot representing mean variant allele frequencies (VAF, in percentage) detected by ddPCR in *SLC35A2*-MOGHE patient FR-2 according to cell subtypes. The ddPCR experiment was performed in separate pools of cells per each category (4 × 250 cortical neurons, 3 × 250 glial cells, 3 × 70 heterotopic neurons and 2 pools of 250 and 500 Olig2-positive cells). Error bars represent the standard deviation. Bulk: whole brain tissue; Glial: glial cells; Glial-Olig2 + : Olig2-positive cells from clusters; Cortical neurons: neurons dissected from the grey matter; Heterotopic neurons: neurons dissected from the white matter

We enrolled 20 sporadic patients with refractory focal epilepsy subjected to neurosurgery (Schoen Klinik, Germany) and postoperatively diagnosed with MOGHE. Amongst subjects, 9/20 had preoperative MRI characteristics of MOGHE as previously described [16]. Clinical and imaging features are summarized in Table 1. The neuropathological diagnosis of MOGHE was in accordance with previously reported MOGHE criteria [35], consisting in the presence of: (1) clusters of increased Olig2-immunoreactive cell density as compared to neighboring regions, close to the grey-white matter junction and deep white matter (Fig. 1a, b); (2) increased densities of heterotopic neurons in the deep white matter according to Mühlebner et al. [28] (Fig. 1c); (3) patchy areas of hypomyelination in the white matter identified by immunohistochemistry for myelin proteins, i.e. CNPase (Fig. 1d).

**Table 1:** Clinical features of the German MOGHE cohort

Patient ID	Sex	Age at seizure onset/Age surgery	Seizure/epilepsy types	MRI findings (1.5T)	Resection topology	Outcome (Engel score)	Follow up after surgery (m)	Previous report
DE-1	M	8m/3y	IS, TS	R frontal FCD	Right frontal	I	63	[16]
DE-2	F	4m/4y	IS	L MOGHE subtype I	Left multilobar	I	39	[16]
DE-3	M	3y4m/4y	IS, TS, MS	R frontal extensive malformation	Right fronto-central	III	23	Unpublished
DE-4	M	7y/13y	TS, MS	R cortical malformation	Right frontal	I	20	Unpublished
DE-5	F	6m/17y	IS, TS, MS	L frontal FCD	Left frontal	IV	17	Unpublished
DE-6	M	18m/6y	IS, TS	L frontal MOGHE subtype II	Left multilobar	I	15	Unpublished
DE-7	F	3m/4y	IS, TS, MS	L frontal MOGHE subtype I	Left frontal	III	13	Unpublished
DE-8	M	1y3m/4y	TS, MS	L frontal MOGHE subtype I	Left fronto-central	I	12	Unpublished
DE-9	F	6m/3y	TS, MS	L frontal MOGHE subtype I	Left frontal	I	6	Unpublished
DE-10	M	6m/4y	IS, TS, MS	L temporo-occipital FCD	Left temporal	IV	81	[16]
DE-11	M	2y/23y	TS, MS	L frontal FCD	Left frontal	I	63	[16]
DE-12	F	5y/10y	TS, MS	L frontal FCD	Left multilobar	I	51	[16]
DE-13	F	1y2m/1y6m	IS	R frontal extensive MOGHE	Right frontal	I	49	[16]
DE-14	M	1y2m/16y	TS	R frontal FCD	Right frontal	I	33	[16]
DE-15	F	6y3m/8y	TS	L frontal FCD	Left frontal	II	30	[16]
DE-16	M	1y9m/10y	IS, TS, MS	L frontal FCD	Left frontal	I	21	Unpublished
DE-17	M	3m/7y	IS, TS	R frontal MOGHE	Right frontal	I	15	Unpublished
DE-18	M	6m/7y	TS	R extensive MOGHE	Right frontal	IV	10	Unpublished
DE-19	M	1y9m/7y	TS, MS	R frontal FCD	Right frontal	NA	5	Unpublished
DE-20	F	3y/4y	TS	L frontal MOGHE	Left frontal	I	5	Unpublished

*F* female, *M* male, *IS* infantile spasms, *TS* tonic seizures, *MS* myoclonic seizures, *L* left, *R* right, *FCD* focal cortical dysplasia, *MOGHE* mild malformation of cortical development with oligodendroglial hyperplasia in epilepsy, *NA* not available

### Genetic findings in the MOGHE cohort

We performed targeted gene panel sequencing in 20 FFPE MOGHE tissues with a mean sequencing coverage of 1161X. We identified somatic *SLC35A2* variants in 9/20 patients (5 males and 4 females), representing a genetic burden of 45% (Table 2). *SLC35A2* variant allele frequencies (VAF) ranged from 7 to 52% (mean VAF: 26.7%). All variants were absent from the gnomAD database [12] (gnomAD v3.1 accessed November 2020). Six loss-of-function variants (frameshift insertions/deletions, nonsense) were considered pathogenic. The three missense variants (p.Thr69Ile, p.Ser302Phe and p.Ser312Phe) were considered likely pathogenic according to ACMG guidelines [34]: presence at a mosaic state (PS2); absence from gnomAD database (PM2); predicted deleterious using two in silico methods, M-CAP, and CADD (all variants scored > 23) and affecting highly conserved amino acids within (Thr69) or in close proximity (Ser302, Ser312) of a helical transmembrane domain (PP3). No matched blood samples were available to confirm the brain specificity of the variants. We did not identify any causative variants in the other panel genes in the 9 *SLC35A2*-positive cases, nor in the 11 *SLC35A2*-negative MOGHE tissue samples.

**Table 2:** *SLC35A2* somatic variants identified in the MOGHE cohort

Patient ID	HGVSc	HGVSp	Variant	Brain VAF (%) Gene panel	Brain VAF (%) Validation	M-CAP	CADD score
DE-1	c.206C > T	p.Thr69Ile	Missense SNV	14	5.75	D	23.8
DE-2	c.603_606dupAGGC	p.Leu203Argfs*20	Fs insertion	21	13.88	–	–
DE-3	c.569_572delGAGG	p.Gly190Alafs*158	Fs deletion	41	47.09	–	–
DE-4	c.335_339dupCGCTC	p.Lys114Argfs*32	Fs insertion	52	Sanger confirmed	–	–
DE-5	c.905C > T	p.Ser302Phe	Missense SNV	7	10.55	D	28.1
DE-6	c.580_616dupCCACTGGATCAGAACCCTGGGGCAGGCCTGGCAGCCG	p.Val206Alafs*28	Fs insertion	9	6.20	–	–
DE-7	c.359_360delTC	p.Leu120Hisfs*7	Fs deletion	30	Sanger confirmed	–	–
DE-8	c.112_116delinsTGGTGGTCCAGAATG	p.Ile38Trpfs*59	Fs indel	33	33.41	–	–
DE-9	c.935C > T	p.Ser312Phe	Missense SNV	33	33.02	D	26.9

All variants were validated by deep amplicon sequencing, except for samples from DE-4 and DE-7, for which Sanger sequencing was used. Patients DE-1 and DE-2 were previously reported and were identified as P8 and P3 respectively in [16]. No blood sample was available to confirm the brain specificity

*Fs* frameshift, *VAF* variant allele frequency, *SNV* single nucleotide variant, *indel* insertion and deletion, *M-CAP* Mendelian clinically applicable pathogenicity score prediction (v1.4), *D* possibly pathogenic, *CADD* combined annotation dependent depletion score (Phred, GRCh37-v1.6). *SLC35A2* RefSeq Transcript: NM_005660

### Neuropathological reassessment of *SLC35A2*-mutated MCD cases

Since MOGHE is a neuropathological entity newly recognized in 2017 [35] and not yet included in the current ILAE classification [4], we asked whether previously identified *SLC35A2*-related MCD cases may be reclassified as MOGHE. To this aim, we assembled an international cohort of 43 MCD cases (including 19 mMCD, 7 FCD1, 9 FCD2, 8 inconclusive diagnosis; see Additional file: Table 1 for histological and genetic cohort description). The cohort included 18 *SLC35A2*-positive cases carrying brain pathogenic variants with an initial diagnosis of mMCD (n = 11), FCD (n = 1) or inconclusive diagnosis (n = 6). A neuropathological reexamination, blind to the initial histological diagnosis and genetic finding, was performed by reviewing all H&E slides, together with anti-NeuN (to identify heterotopic neurons) and anti-Olig2 (to identify clusters of oligodendroglial cells) immunostainings. In 17 out of 18 cases with an *SLC35A2*-variant, we confirmed the microscopic signature of MOGHE (Table 3) with clusters of increased Olig2-positive cells at the grey-white matter junction and deep white matter, and increased densities of heterotopic neurons in the white matter. In one *SLC35A2* case, the brain specimen was too fragmented to allow a meaningful interpretation. No MOGHE diagnosis was established for non-*SLC35A2*-mutated cases. This observation is in support of a homogenous neuropathological phenotype associated with somatic *SLC35A2* variants. Clinical features of reassessed *SLC35A2*-MOGHE cases are presented in Table 4.

**Table 3:** Neuropathological reassessment of *SLC35A2*-mutated case

Patient ID	HGVSc	HGVSp	CADD score	Brain VAF (%)	Initial histology	Histology reclassified	Previous report
FR-1	c.801C > G	p.Tyr267*	35	12.1	mMCD2, blurred grey-white matter border*	MOGHE	[2]
FR-2	c.634_635delTC	p.Ser212Leufs*9	–	2–22.6	mMCD2, blurred grey-white matter border, increased oligodendrocytes density*	MOGHE	[2]
FR-3	c.886_888delCTC	p.Leu296del	–	22.4	mMCD2, blurred grey-white matter border*	MOGHE	[2]
FR-4	c.804dupA	p.Pro269Thrfs*25	–	32.7	mMCD2, blurred grey-white matter border, increased oligodendrocytes density, white matter pallor*	MOGHE	[2]
FR-5	c.918_929delGCTGTCCACTGT	p.Leu307_Val310del	–	13	FCD unclassified	MOGHE	Unpublished
FR-6	c.287_288delAC	p.His96Profs*7	–	25	Not conclusive (fragmented tissue)	Not conclusive (fragmented tissue)	Unpublished
KR-1	c.703A > C	p.Asn235His	25.8	10	mMCD	MOGHE	[38]
KR-2	c.275-1G > T	–	35	5	Gliosis	MOGHE	[38]
KR-3	c.553C > T	p.Gln185*	35	6	No abnormality	MOGHE	[38]
KR-4	c.760G > T	p.Glu254*	37	15.8	mMCD	MOGHE	[38]
KR-5	c.589C > T	p.Gln197*	36	22.9	mMCD	MOGHE	[38]
KR-6	c.502C > T	p.Gln168*	36	18	mMCD	MOGHE	[38]
KR-7	c.359T > C	p.Leu120Pro	27.4	1.4	Gliosis	MOGHE	[39]
KR-8	c.842G > A	p.Gly281Asp	23.4	3.7	Gliosis	MOGHE	[39]
KR-9	c.671T > C	p.Leu224Pro	27.5	3.7	Gliosis	MOGHE	[39]
KR-10	c.359_360delTC	p.Leu120Hisfs*7	–	5.5	mMCD	MOGHE	Unpublished
NL-1	c.385C > T	p.Gln129*	36	33	mMCD2	MOGHE	Unpublished
NL-2	c.553C > T	p.Gln185*	35	14.5	mMCD2	MOGHE	Unpublished

*Patients with an initial histological diagnosis suggestive of MOGHE. Patient FR-2 underwent three surgeries, and we confirmed the presence of a mosaic gradient, with a 2% VAF identified in the tissue from the first surgery and a 22.6% VAF in the tissue from the second surgery. Tissue from the third surgery was not tested for the genetic variant

*SLC35A2* variants were absent from blood-derived DNA when available (all except KR-10, NL-1, NL-2)

**Table 4:** Clinical features of reassessed *SLC35A2*-MOGHE patients

Patient ID	Sex	Age at seizure onset/age surgery	Seizure/epilepsy types	MRI findings (3T)	Resection topology	Outcome (Engel score)	Follow up after surgery (m)	Previous reports
FR-1	M	1y6m/3y	IS	L frontal extensive FCD	Left frontal	I	30	[2]
FR-2	M	7m/4y	IS	R temporal FCD	Right multilobar	III	33	[2]
FR-3	M	2y6m/8y	IS	L temporo-occipital FCD	Left multilobar	I	9	[2]
FR-4	M	5m/3y	IS	R frontal extensive FCD	Right multilobar	I	15	[2]
FR-5	F	6m/13y	IS, LGS	L fronto-basal FCD	Left multilobar	I	12	Unpublished
KR-1	M	3m/3y5m	IS, LGS	MRI-negative*	Right multilobar	IV	49	[38]
KR-2	M	3m/5y2m	IS, LGS	MRI-negative*	Left multilobar	I	75	[38]
KR-3	F	13m/5y1m	IS, LGS	MRI-negative*	Left multilobar	I	69	[38]
KR-4	M	3y2m/6y3m	IS, LGS	MRI-negative*	Left multilobar	I	57	[38]
KR-5	F	5m/4y	IS, LGS, MS	R frontal FCD	Right multilobar	I	32	[38]
KR-6	M	7m/2y8m	IS, LGS	R temporo-parietal increase white matter signal in T2	Right parietal	I	26	[38]
KR-7	M	6m/2y1m	IS	MRI-negative*	Right multilobar	I	48	[39]
KR-8	M	2y6m/6y1m	LGS	Left frontal FCD	Left frontal	I	30	[39]
KR-9	F	10m/10y2m	LGS	MRI-negative*	Left multilobar	IV	30	[39]
KR-10	F	6m/9y	IS, LGS	MRI-negative*	Right temporal	I	11	Unpublished
NL-1	M	NA/5y	Focal to bilateral TCS	MRI-negative*	Left occipital	I	24	Unpublished
NL-2	F	NA/1y	IS	L extensive cortical malformation	Left frontal	I	12	Unpublished

*F* female, *M* male, *IS* infantile spasms, *TS* tonic seizures, *MS* myoclonic seizures, *LGS* Lennox-Gastaut syndrome, *TCS* tonic–clonic seizures, *L* left, *R* right, *FCD* focal cortical dysplasia, *NA* not available

*Different MRI protocols were used among the centers, therefore MRI-negative cases should be interpreted with caution

### Clinical and genetic landscape of *SLC35A2*-mutated MOGHE

Altogether, we report a series of 26 sporadic MOGHE subjects with a brain somatic *SLC35A2* variant (n = 9 from the German cohort described in this study and n = 17 histologically reassessed cases from France, Republic of Korea and the Netherlands). All patients had refractory focal epilepsy and were subjected to surgery. The whole cohort comprised 16 males and 10 females representing a sex-ratio M/F of 1.6. Age at epilepsy onset ranged from 3 months to 7 years (mean: 1 year and 3 months). Infantile spams were reported in 77% (20/26) and were commonly associated with tonic seizures in 66% (12/18) of cases, while clonic seizures were reported in 31% (8/26) of cases. Impaired awareness during the seizure was reported in 50% (13/26) of patients. All patients (26/26) experienced daily seizures that occurred both during sleep and wake. Neurological evaluation was normal in 85% (22/26) of cases. Multiple epileptic foci were detected on presurgical electroencephalography (EEG) in more than one-third of cases (40%, 10/25). Presurgical MRI was interpreted with a focal cortical abnormality suggestive of MCD in 69% (18/26) of the entire cohort. A fraction of patients (DE-1 to DE-9) were specifically assessed for previously reported MOGHE MRI characteristics [16]. Fluoro-deoxyglucose (FDG) positron emission tomography (PET) was performed in 46% (12/26) of patients and showed a focal cortical interictal hypometabolism in nearly all patients (11/12). Among them, PET hypometabolism was concordant with the ictal EEG region in 82% (9/11) cases. A cognitive impairment was reported before surgery in almost all cases (24/26) showing a mild to moderate delay in 63% (15/24) and a severe delay in 35% (9/24) of them. Age at surgery ranged from 1 to 17 years (mean: 5 years and 10 months) and four patients underwent at least two surgeries to achieve seizure control. A frontal lobe resection was most frequently performed and concerned 81% (21/26) of surgical procedures. Patients were post-surgically followed up for 6 months to 6.3 years ($\ge$ 2 years for 14 patients). According to the Engel Epilepsy Surgery Outcome Scale, 77% (20/26) of cases were seizure-free or had only rare disabling seizures (Engel I–II, mean follow-up: 2.7 years), while 23% (6/26) of cases had no improvement or a worthwhile improvement (Engel III–IV, mean follow-up: 2.5 years).

In total, we reported 24 distinct brain somatic variants (Fig. 2). Among the 26 patients, five had a recurrent somatic pathogenic *SLC35A2* variant. Loss-of-function variants were the most frequent with 37% frameshift insertions/deletions, 25% nonsense SNV and 4% splice site SNV. Missense variants (30%) and in-frame insertions/deletions (4%) were considered as likely pathogenic based on in silico pathogenicity prediction, protein domain localization, amino acid conservation among species, absence from the gnomAD database and their presence at mosaic state in the lesional tissue. The variants p.Ser212Leufs*9, p.Leu120Hisfs*7 and p.Gln185* were recurrent somatic variants; p.Ser212Leufs*9 was previously reported in two other sporadic cases with focal epilepsy related to MCD [25, 41]. *SLC35A2* variant allele frequencies (VAFs) ranged from 1.4 to 52% among the cases.

To determine possible genotype–phenotype correlations, we compared clinical features and surgery outcome between MOGHE *SLC35A2*-mutated cases (n = 26) and panel-negative cases (n = 11) (Additional file: Table 2). We did not observe significant differences in age at seizure onset, duration of epilepsy before surgery, neurodevelopmental status and post-surgical outcome. The only feature distinguishing *SLC35A2* cases from panel-negative cases was a higher proportion of infantile spasms in *SLC35A2*-MOGHE cases (77% vs. 36%, *p* = 0.01).

### Oligodendroglial cells and heterotopic neurons carry *SLC35A2* variants

We next asked which are the specific cell types carrying *SLC35A2* variants. To address this question, by laser capture microdissection we isolated glial cells located in the white matter, heterotopic neurons at the blurred grey-white matter junction and morphologically normal neurons within the grey matter, based on their morphology on cresyl-violet stained frozen sections from one male MOGHE case (FR-2, VAF in the bulk brain tissue of 22.6%). Variant allele frequency was determined in each pool by droplet digital PCR. Since *SLC35A2* is an X-linked gene, present in one copy in males, the VAF directly reflects the percentage of cells carrying the variant. We found a VAF enrichment in both pools of glial cells (36.5 ± 6.2%) and heterotopic neurons (28.6 ± 4.2%) compared to morphologically normal neurons (8.7 ± 3.1%) (Fig. 3). These results indicate that both glial cells and heterotopic neurons, rather than morphologically normal cortical neurons are the main carriers of *SLC35A2* variants in MOGHE. We then hypothesized that clusters of high Olig2-positive cells density have a higher VAF as a consequence of the *SLC35A2* variant. Analysis of microdissected pools of Olig2-positive cells located in clusters within the white matter showed that nearly half of these Olig2-positive cells carried the variant (mean VAF = 50.5 ± 18%). Altogether, these results provide the proof of principle demonstration that *SLC35A2* variants are enriched in pathological cell types featuring MOGHE.

## Discussion

**Fig. 4:**
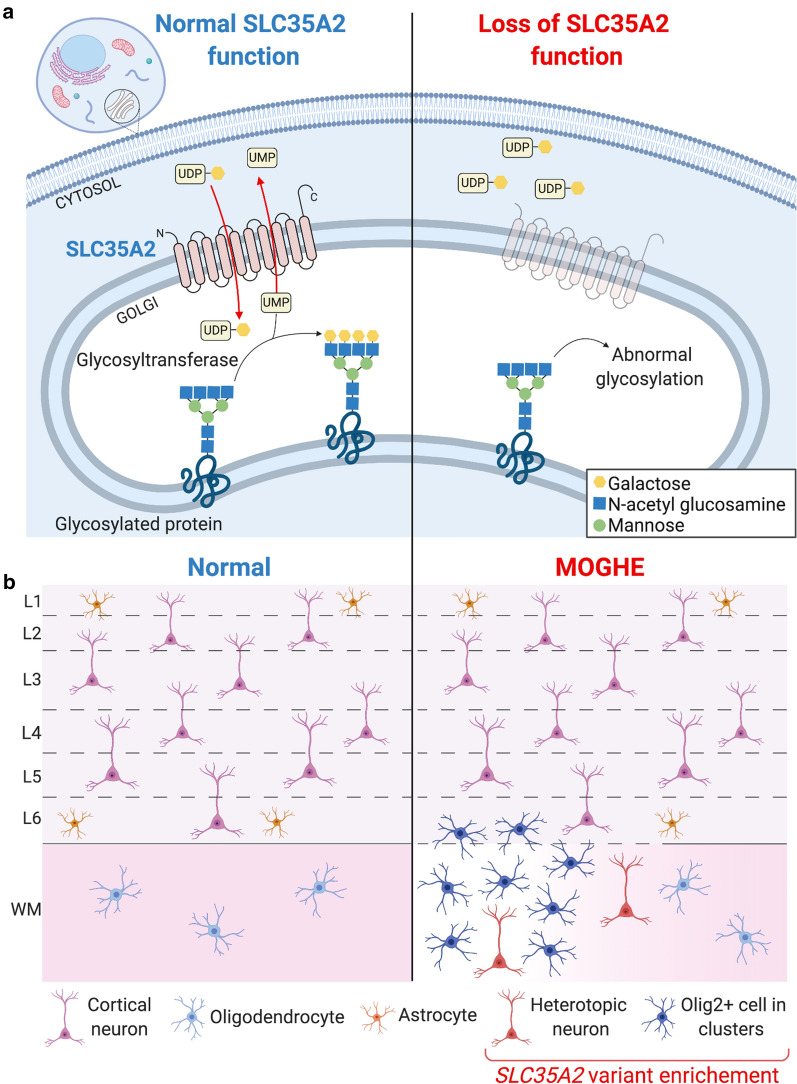
Simplified representation of pathophysiology and neuropathology findings in *SLC35A2*-related MOGHE cases. **a** *SLC35A2* loss-of-function variants (right) cause a defect in protein/sphingolipid glycosylation in the cell, with reduced galactose residues due to the diminished UDP-galactose availability within the Golgi apparatus and endoplasmic reticulum. **b** Loss of SLC35A2 function leads to a MOGHE phenotype, with clusters of increased density of Olig2-positive cells in the white matter and deep cortical layers, a blurred grey-white matter boundary (dotted line), patchy hypomyelination (color gradient within the white matter) and heterotopic neurons in the white matter. Analysis of microdissected cells revealed that *SLC35A2* pathogenic variant is enriched in oligodendroglial cells isolated from clusters with increased cell density and heterotopic neurons. L1-L6: layers 1-6; WM: myelination in the white matter; *Olig2+* Olig2-positive cells

In this study we report the so far largest cohort of patients with MOGHE, a newly recognized clinico-pathological entity belonging to the spectrum of epileptogenic mild malformations of cortical development (mMCD). Here we took advantage of advanced deep sequencing technology and bioinformatic tools to detect brain somatic variants. We used unmatched FFPE tissues, the most common archival brain tissue source and highlighted the importance of genetic diagnosis for comprehensive genotype–phenotype studies. Our findings demonstrate that somatic brain-only variants in the UDP-galactose transporter gene *SCL35A2* are a frequent cause in MOGHE. Most *SCL35A2* variants are nonsense or frameshift variants leading to loss-of-function of the protein in the mutated cells (Fig. 4a). Pathogenicity of three *SLC35A2* variants reported in this study has been demonstrated in previous studies: (1) Sim et al. showed aberrant pattern of glycosylation in tissues carrying the *SLC35A2* brain somatic variants p.Glu254* and p.Gln197* (patients KR-4 and KR-5 in this study respectively) [38]; (2) Ng et al. performed a biochemical assay on a *SLC35A2*-CDG patient fibroblasts carrying the p.Gln168* variant (same variant here described in patient KR-6) to confirm that SLC35A2-dependent UDP-galactose transport into the Golgi apparatus is altered [30].

Diagnosis of MCD remains challenging in everyday clinical practice [32] and white matter lesions in the borderland of FCD are lacking a comprehensive definition and description of clinical phenotypes, imaging features or specific molecular biomarkers [29]. MOGHE is currently not included in the 2011 ILAE classification of FCD [4], since it was first described histopathologically in 2017 [35] and is therefore likely underdiagnosed in current clinical practice. By reevaluating previously diagnosed *SLC35A2* cases including anti-Olig2 immunostainings, we confirmed they all belong to the MOGHE entity. We next asked whether histopathological hallmarks of MOGHE comprising clusters of increased Olig2-immunoreactive cells, patchy areas of hypomyelination and foci of heterotopic neurons in the white matter were a direct consequence of the *SLC35A2* variants. By means of a single cell approach performed in a MOGHE tissue, we identified that the *SLC35A2* variant was enriched in both Olig2-positive cell clusters and heterotopic neurons (Fig. 4b), suggesting that the mutational event occurred during brain development in a neuroglial progenitor cell, likely in radial glia cells, before the differentiation into neuronal and glial cell lineages.

Protein N-glycosylation is a key post-translational process, and the majority of all secretory glycoproteins appear to be N-glycosylated. Altered glycan structures may affect folding, stability, interaction with other molecules, and cell-surface expression of numerous proteins. Studies of CDG which manifest with severe neurological symptoms have highlighted the importance of N-glycosylation on brain development [11, 33]. There is evidence that N-glycans contribute to the regulation of neural transmission and excitability of neural circuits [36], and in neuronal adhesion and neuronal migration [24] due to altered extracellular matrix proteoglycans [1]. Glycosylation can also affect the myelination process due to abnormal development of myelin sheaths constituted by glycolipids or abnormal connections between oligodendrocytes and their myelin sheath [6, 15, 17]. This hypothesis is further sustained by the report of myelination defects in other congenital disorders of glycosylation [11, 33]. It remains to be further investigated how a primary defect in glycosylation in neuroglial progenitors leads to the histopathological features described in MOGHE.

Patients with *SLC35A2*-related MOGHE have intractable focal epilepsy; however the underlying pathomechanisms remain unclear. In particular, while heterotopic neurons interconnected within neuronal networks may drive the epileptogenicity, to which extent the oligodendroglial hyperplasia and/or hypomyelination contribute to epileptogenesis is unknown. To further clarify these mechanisms, *SLC35A2*-deficient mouse models will be needed to dissect the spatio-temporal contribution of each cell type. Accordingly, a recent study reported a correlation between interictal EEG spike density and the *SLC35A2* variant allele burden from different brain areas of a patient who underwent multilobar resection [25]. In FCD type 2 and hemimegalencephaly linked to mosaic variants in genes of the mTOR pathway, a similar correlation between the size of the lesion or the epileptogenic activity and the mosaic rate has also been reported confirming the pro-epileptogenic role of mutated cells [2, 20, 21].

The finding of *SLC35A2* variants as a cause of MOGHE may also promote the development of precision therapies. Two reports showed a clinical improvement in response to oral D‐galactose supplementation in *SLC35A2*-CDG patients, resulting in reduction of seizure frequency [10, 42]. Galactose supplementation may partially increase cytosolic UDP-galactose, thus facilitating galactosylation through alternative UDP-galactose transport into the Golgi [14, 22]. Evaluating this therapeutic approach in patients with brain mosaic loss of *SLC35A2*, and for whom surgery is not an option or failed to reduce seizures, could offer a personalized treatment strategy. Regarding the 55% (11/20) *SLC35A2*-negative MOGHE cases of the cohort, the causal gene still needs to be identified, either by targeted sequencing of genes belonging to other glycosylation pathways or by deep whole-exome/genome sequencing.

## Conclusion

Our findings highlight the important contribution of brain mosaicism in the etiology of focal epilepsies associated with MCDs. FCD type 2 are well recognized as mosaic mTORopathies, due to brain somatic variants in the PI3K-AKT3-mTOR pathway [2, 8, 26, 39]. Here we confirm that MOGHE is a brain mosaic disorder affecting the N-glycosylation pathway. Our work further emphasizes the importance of genetic diagnosis in neuropathologically characterized human brain tissues to provide guidance toward precision therapeutic targets in these refractory focal epilepsies.

## Supplementary information

**Additional file: 1**. Supplemental data. Full list of genes included in the panel. SupplementaryTable 1. Neuropathological reassessment of multicentric cohort of 43 MCD cases. Supplementary Table 2. Clinical comparisons between SLC35A2-mutated and panelnegative MOGHE cases.

## Abbreviations

MOGHE: mild malformation of cortical development with oligodendroglial hyperplasia in epilepsy
MCD: malformations of cortical development, mMCD: mild malformations of cortical development
FCD: focal cortical dysplasia
MRI: magnetic resonance imaging
FLAIR: fluid attenuated inversion recovery
mTOR: mechanistic Target of Rapamycin
CDG: congenital disorder of glycosylation
EEG: electroencephalograpy
PET: positron emission tomography
FDG: fluoro-deoxy-glucose
FFPE: formalin-fixed paraffin-embedded
SNV: single nucleotide variant
M-CAP: mendelian clinically applicable pathogenicity
CADD: combined annotation-dependent depletion
LCM: laser capture microdissection
ddPCR: droplet digital polymerase chain reaction
VAF: variant allele frequencies
indel: insertion and/or deletion
ILAE: International League Against Epilepsy

## Availability of data and materials

All data generated or analyzed during this study are included in this published article (and its supplementary information files).
