# Supplementary material for: Frequent SLC35A2 brain mosaicism in mild malformation of cortical development with oligodendroglial hyperplasia in epilepsy (MOGHE)
Source: Acta Neuropathol Commun. 2021 Jan 6;9:3. doi: 10.1186/s40478-020-01085-3 (PMC7788938; doi:10.1186/s40478-020-01085-3)
Supplement: Supplementary file 1 — Additional file: 1. Supplemental data. Full list of genes included in the panel. SupplementaryTable 1. Neuropathological reassessment of multicentric cohort of 43 MCD cases. Supplementary Table 2. Clinical comparisons between SLC35A2-mutated and panelnegative MOGHE cases. [file 40478_2020_1085_MOESM1_ESM.pdf]

Frequent *SLC35A2* brain mosaicism in mild malformation of cortical development with oligodendroglial hyperplasia in epilepsy (MOGHE)

Thomas Bonduelle[1], Till Hartlieb[2,3], Sara Baldassari[1], Nam Suk Sim[4], Se Hoon Kim[5], Hoon-Chul Kang[6], Katja Kobow[7], Roland Coras[7], Mathilde Chipaux[8], Georg Dorfmüller[8], Homa Adle-Biassette[9], Eleonora Aronica[10,11], Jeong Ho Lee*[4,12], Ingmar Blumcke*[7] and Stéphanie Baulac*[1]

1 Sorbonne Université, Institut du Cerveau - Paris Brain Institute - ICM, Inserm, CNRS, F-75013, Paris, France
2 Center for pediatric neurology, neurorehabilitation and epileptology, Schoen Klinik, Vogtareuth, Germany
3 Paracelsus Medical University, Salzburg, Austria
4 Graduate School of Medical Science and Engineering, KAIST, Daejeon, Korea
5 Department of Pathology, Yonsei University College of Medicine, Seoul, Korea
6 Division of Pediatric Neurology, Department of Pediatrics, Pediatric Epilepsy Clinics, Severance Children's Hospital, Yonsei University College of Medicine, Seoul, Korea
7 Department of Neuropathology, Universitätsklinikum Erlangen, Friedrich-Alexander University (FAU) Erlangen-Nürnberg, Erlangen, Germany
8 Department of Pediatric Neurosurgery, Rothschild Foundation Hospital, F-75019 Paris, France
9 Service d'Anatomie et de Cytologie Pathologiques, Hôpital Lariboisière, APHP, 75010, Paris, France
10 Amsterdam UMC, University of Amsterdam, Department of (Neuro)Pathology, Amsterdam Neuroscience, Amsterdam, the Netherlands
11 Stichting Epilepsie Instellingen Nederland (SEIN), Heemstede, the Netherlands
12 SoVarGen, Inc., Daejeon 34051, Republic of Korea.
* equal contribution

Corresponding author:
Stéphanie Baulac, PhD
Institut du Cerveau - ICM
Hôpital Pitié-Salpêtrière - 47, bd de l'hôpital - 75013 Paris
Phone : +33 (0)1 57 27 43 39 / E-mail: stephanie.baulac@icm-institute.org

SUPPLEMENTARY FILES

Supplemental data Full list of genes included in the panel

PIK3CA (NM_006218), *PIK3R2* (NM_005027), *PIK3R1* (NM_181523), *PTEN* (NM_000314), *AKT1* (NM_005163), *AKT2* (NM_001626), *AKT3* (NM_005465), *GSK3B* (NM_002093), *GSK3A* (NM_019884), *MTOR* (NM_004958), *TSC1* (NM_000368), *TSC2* (NM_000548), *TBC1D7* (NM_016495), *DEPDC5* (NM_001242896), *NPRL2* (NM_006545), *NPRL3* (NM_001077350), WDR24 (NM_032259), MIOS (NM_019005), SEC13 (NM_183352), *WDR59* (NM_030581), *SEH1L* (NM_001013437), *RHEB* (NM_005614), *RHEBL1* (NM_144593), *RRAGA* (NM_006570), *RRAGB* (NM_016656), *RRAGC* (NM_022157), *RRAGD* (NM_021244), *BMT2* (NM_152556), *DEPTOR* (NM_022783.3), *RPTOR* (NM_020761), *MLST8* (NM_001199173.1), *AKT1S1* (NM_032375), *RICTOR* (NM_001285439.1), *PRR5* (NM_001198721), *PRR5L* (NM_024841), *MAPKAP1* (NM_001006617), *PDPK1* (NM_002613), *RPS6KB1* (NM_003161.3), *RPS6KB2* (NM_003952), *SESN1* (NM_014454), *SESN2* (NM_031459), *SESN3* (NM_144665.3), *CASTOR1* (NM_001037666), *CASTOR2* (NM_001145064), *FLCN* (NM_144997), *KPTN* (NM_007059), *SZT2* (NM_015284.3), *ITFG2* (NM_018463), *C12ORF66* (NM_001300940), DDIT4 (NM_019058), *LAMTOR1* (NM_017907), *LAMTOR2* (NM_014017), *LAMTOR3* (NM_021970), *LAMTOR4* (NM_001008395.3), *LAMTOR5* (NM_006402), *SLC38A9* (NM_173514), *SLC35A2* (NM_005660.2), *KRT1* (NM_006121), *KRT10* (NM_000421), *SRY* (NM_003140).

Supplementary Table 1 Neuropathological reassessment of multicentric cohort of 43 MCD cases

Patient ID	Initial histology	Histology reclassified	Genetic findings	Previous report
FR-1	mMCD2, blurred grey-white matter border*	MOGHE	SLC35A2:p.Tyr267*	Baldassari *et al.* 2019
FR-2	mMCD2, blurred grey-white matter border, increased oligodendrocytes density*	MOGHE	SLC35A2:p.Ser212Leu*fs**9	Baldassari *et al.* 2019
FR-3	mMCD2, blurred grey-white matter border*	MOGHE	SLC35A2:p.Leu296del	Baldassari *et al.* 2019
FR-4	mMCD2, blurred grey-white matter border, increased oligodendrocytes density, white matter pallor*	MOGHE	SLC35A2:p.Pro269Thr*fs**25	Baldassari *et al.* 2019
FR-5	FCD unclassified	MOGHE	SLC35A2:p.Leu307_Val310del	unpublished
FR-6	Not conclusive (fragmented tissue)	Not conclusive (fragmented tissue)	SLC35A2: p.His96Pro*fs**7	unpublished
FR-7	mMCD2	mMCD	Panel-negative	Baldassari *et al.* 2019
FR-8	mMCD2	mMCD	Panel-negative	Baldassari *et al.* 2019
FR-9	mMCD2	mMCD	Panel-negative	Baldassari *et al.* 2019
FR-10	mMCD2	MCD with gliosis and possible sEEG-induced infarction	Panel-negative	Baldassari *et al.* 2019
FR-11	FCD1b	MCD with possible sEEG-induced infarction	Panel-negative	Baldassari *et al.* 2019
FR-12	FCD1	FCD3d with L4 necrosis	Panel-negative	Baldassari *et al.* 2019

FR-13	FCD1a	FCD1a	Panel-negative	Baldassari *et al.* 2019
FR-14	FCD1a	Likely mMCD	Panel-negative	Baldassari *et al.* 2019
FR-15	mMCD2	mMCD	Panel-negative	unpublished
FR-16	FCD1b	HS type I and mMCD	Panel-negative	Baldassari *et al.* 2019
FR-17	mMCD2	mMCD	Panel-negative	Baldassari *et al.* 2019
FR-18	FCD1a	No HS, temporal cortex	Panel-negative	Baldassari *et al.* 2019
FR-19	FCD1	Ulegyria or HET	Panel-negative	Baldassari *et al.* 2019
FR-20	mMCD2	Infarction	Panel-negative	Baldassari *et al.* 2019
NL-1	mMCD2	MOGHE	SLC35A2:p.Gln129*	unpublished
NL-2	mMCD2	MOGHE	SLC35A2:p.Gln185*	unpublished
NL-3	FCD2a	FCD 2a	DEPDC5:p.Thr1385*fs*	unpublished
NL-4	FCD2a	FCD 2a	DEPDC5:p.Ala1386*fs*	unpublished
NL-5	FCD2a	FCD 2a	DEPDC5:p.Asn1353*fs*	unpublished
NL-6	FCD2a	FCD 2a	DEPDC5:p.Gly756*fs*	unpublished
KR-1	mMCD	MOGHE	SLC35A2:p.Asn235His	Sim *et al.* 2018
KR-2	Gliosis	MOGHE	*SLC35A2*:c.275-1G>T	Sim *et al.* 2018
KR-3	No abnormality	MOGHE	SLC35A2:p.Gln185*	Sim *et al.* 2018
KR-4	mMCD	MOGHE	SLC35A2:p.Glu254*	Sim *et al.* 2018
KR-5	mMCD	MOGHE	SLC35A2:p.Gln197*	Sim *et al.* 2018
KR-6	mMCD	MOGHE	SLC35A2:p.Gln168*	Sim *et al.* 2018
KR-7	Gliosis	MOGHE	SLC35A2:p.Leu120Pro	Sim *et al.* 2019
KR-8	Gliosis	MOGHE	SLC35A2:p.Gly281Asp	Sim *et al.* 2019
KR-9	Gliosis	MOGHE	SLC35A2:p.Leu224Pro	Sim *et al.* 2019
KR-10	mMCD	MOGHE	SLC35A2:p.Leu120His*fs**7	unpublished

KR-11	FCD2a	FCD2a	DEPDC5:p.Gln372*	Sim *et al.* 2019
KR-12	FCD2b	FCD2a	DEPDC5:p.Phe1399*fs*<br>DEPDC5:p.Thr1508*fs*	Sim *et al.* 2019
KR-13	FCD2a	FCD2a	DEPDC5:p.Trp1213*	Sim *et al.* 2019
KR-14	FCD2a	FCD2a	DEPDC5:p.Arg1268*	Sim *et al.* 2019
KR-15	mMCD	FCD2a	*DEPDC5*:c.3021+1G>A	Sim *et al.* 2019
KR-16	FCD2a	FCD2a	DEPDC5:p.Arg1136*	Sim *et al.* 2019
KR-17	No abnormality	Inconclusive	DEPDC5:c.1324+1G>A	Sim *et al.* 2019

GM: grey matter; WM: white matter; mMCD: mild Malformations of Cortical Development; FCD: Focal Cortical Dysplasia; sEEG: stereo EEG; HS: Hippocampal Sclerosis; HET: Heterotopia.

Supplementary Table 2 Clinical comparisons between SLC35A2-mutated and panel-negative MOGHE cases

	**MOGHE *SLC35A2*-mutated cases (n=26)**	**MOGHE without variant identified (n=11)**	**p-value**
**Age at epilepsy onset** (years, mean)	1.3 [0.25-7]	2.1 [0.25-6.25]	0.13
**Duration of epilepsy before surgery** (years, mean)	4.4 [1-16.7]	6.9 [0.33-21.8]	0.29
**Infantile spasms** (%)	77% (n=20/26)	36% (n=4/11)	**0.01**
**Tonic seizures** (%)	66% (n=12/18)	90% (n=10/11)	0.13
**Myoclonic seizures** (%)	31% (n=8/26)	45% (n=5/11)	0.39
**Autistic features** (%)	31% (n=8/24)	27% (n=3/11)	0.72
**Developmental delay** (including mild, moderate and severe) (%)	92% (n=24/26)	90% (n=10/11)	0.88
**Good post-operative outcome** (Engel I-II, %)	77% (n=20/26)	80% (n=8/10)	0.84
**Poor post-operative outcome** (Engel III-IV, %)	23% (n=6/26)	20% (n=2/10)	0.84
**Multiple surgeries** (%)	15% (n=4/26)	9% (n=1/11)	0.6

MOGHE *SLC35A2*-mutated cohort (n=26) was obtained associating German *SLC35A2*-MOGHE cases identified in this study (n=9) together with multicentric histologically reassessed *SLC35A2*-MOGHE cases (n=5 from France, n=10 from Republic of Korean and n=2 from the Netherlands). MOGHE patients without variant identified (n=11) correspond to panel-negative German MOGHE cases. All percentages are presented based on each available patient's characteristics. Statistical analysis were performed using Chi-2 test for percentages comparisons and Wilcoxon test for mean comparisons.
